# Preanalytical errors in emergency department samples: Investigating error sources

**DOI:** 10.5937/jomb0-25263

**Published:** 2020-10-02

**Authors:** Adolfo Romero, Juan Gómez-Salgado, Adolfo Romero-Arana, José Antonio Gómez-Fernández, Andrés Cobos, María Carmen Ramos, María Rosa Iglesias

**Affiliations:** 1 University of Málaga, Health Sciences School, University Hospital Virgen de la Victoria, Nursing and Podiatry Department, Málaga, Spain; 2 University of Huelva, University School of Social Work, Department of Sociology, Social Work and Public Health, Huelva, Spain; 3 Universidad Espíritu Santo, Safety and Health Postgraduate Programme, Guayaquil, Ecuador; 4 Institute of Biomedical Research of Málaga (IBIMA), Málaga, Spain; 5 University Hospital Juan Ramón Jiménez, Laboratory Department, Huelva, Spain; 6 University Hospital Virgen de la Victoria, Laboratory Department, Málaga, Spain; 7 University of Málaga, Health Sciences School, Nursing and Podiatry Department, Málaga, Spain

**Keywords:** emergency hospital service, hospital laboratory, preanalytical phase errors, qualitative research, kvalitativna istraživanja, greške u preanalitičkoj fazi, bolnička laboratorija, urgentna služba

## Abstract

**Background:**

The presence of preanalytical errors is a recurring fact in all areas of healthcare that send samples to laboratories. Increasing the knowledge of possible sources of error in the preanalytical phase has been the objective of this group during the last 10 years.

**Methods:**

In this study, descriptive research has been carried out using professionals' opinions obtained by means of the Strengths, Weaknesses, Opportunities, and Threats method in a focus group.

**Results:**

The opinions expressed within the focus group have emphasised the importance of patients' safety and willingness for the introduction of a computerized analytical module. The most commented weakness in both hospitals was the transport of samples through the pneumatic tube. Improving the duration of workers' contracts, especially in the laboratory, and creating a circuit for professional's localization during the work shift to facilitate potential error solving are some opportunities for the future.

**Conclusions:**

Different approaches have been developed depending on the healthcare scenario. For this, establishing a flow of information between the different professionals allows identifying identical aspects through a priori, different points of view. The line to follow is to improve the safety of the patient and also to give professionals an opportunity to express themselves.

## Abstract (sr)

**Uvod:**

Preanalitičke greške se javljaju u radu svih delova sistema zdravstvene zaštite koji šalju uzorke u laboratorije. Cilj ove grupe tokom poslednjih 10 godina je bio povećanje znanja o mogućim izvorima grešaka u preanalitičkoj fazi.

**Metode:**

Ova studija je sprovela deskriptivno istraživanje zasnovano na zapažanjima zaposlenih koristeći SWOT analizu u fokus grupi.

**Rezultati:**

Mišljenja koja su izražena u fokus grupi su potcrtala važnost bezbednosti pacijenata kao i postojanje volje za uvođenjem kompjuterizovanog analitičkog modula. Nedostatak koji se najviše isticao u obe bolnice odnosio se na transport uzoraka kroz pneumatsku cev. Poboljšanja u vezi sa trajanjem ugovora zaposlenih, posebno u laboratoriji, i stvaranje okruženja u kome bi uposleni mogli da se oslone jedni na druge u toku radne smene kako bi se olakšalo potencijalno rešavanje grešaka su neke od stvari koje treba razmotriti u budućnosti.

**Zaključak:**

Postoje različiti pristupi u zavisnosti od vrste zdravstvene zaštite. Uspostavljanje protoka informacija između različitih delova i činioca sistema omogućava prepoznavanje identičnih aspekata sa različitih stanovišta. Ono što svakako treba poboljšati to je bezbednost pacijenata i pružiti priliku profesionalcima da iskažu svoje mišljenje.

## Introduction

The presence of preanalytical errors is a recurring fact in all areas of healthcare that send samples to laboratories. In particular, they account for over 60% of the detected errors, so establishing strategies that foster prevention has been considered a preferred action in any health policy [1].

One of the possible areas of occurrence is the stat laboratory where, especially in large hospitals, a high number of samples are received from an emergency department (ED), obtained in many cases from critically ill patients and under conditions of stress. In this sense, the need to request routine testing which provides little information to the clinician but increases both the workload and the health expenditure [2] [3] has been discussed, previously finding that the accessibility of these actions is not equal in all health centres [4]. This provided factual information in a context in which variations (about technical procedures and workflows) can be important, given that a large number of variables act as an influence.

One of the most decisive aspects this line of research has shown is that in rare occasions are professionals involved in different processes consulted, something that often results in a substantial deficit of knowledge of the actual environment in which they operate.

A highly recommended option is to use the qualitative methodology (focus group), following the development scheme of the analysis of strengths, weaknesses, opportunities, and threats (SWOT). In this sense, we applied the experiences shared by the staff involved in the preanalytical process in both primary care and routine laboratory during the design stage of this new study with the professionals who worked with ED samples [5] [6].

Various authors have also developed an HFMEA (Healthcare Failure Mode and Effects Analysis) that allows for improving the capability of errors detection in samples provided by primary care, with good results both in the detection of errors areas as well in the involvement of professionals [7].

We thus intend in this article to increase knowledge about the possible error sources into the preanalytical phase in ED samples, obtaining opinions by professionals that order, obtain and process urgent samples, as a complementary tool to enhance the capability of errors detection.

## Materials and Methods

### Subjects

A descriptive qualitative study through the focal group technique and following the outline of the SWOT analysis was done at two centres of our regional health system (Virgen de la Victoria Hospital in Málaga and Juan Ramón Jiménez Hospital, in Huelva). The study was performed in May 2017.

At the University Hospital Virgen de la Victoria (HUVV), eight professionals were intentionally selected, and six of them finally attended the session. All of them were involved in the preanalytical phase process at the emergency department and the stat laboratory (a resident doctor, a resident pharmacist, two ED nurses, and two laboratory technicians).

At the University Hospital Juan Ramón Jiménez (HJRJ), seven professionals were also intentionally selected and participated in the study (an emergency senior register, a laboratory senior register responsible for the emergency laboratory, two ED nurses, two laboratory technicians, and an ED auxiliary nurse).

The selection of these professionals followed two inclusion criteria: the capacity for transmission of information on the research topic, and social significance in the sense of being considered key players in the process. Both the workplace and the age were distributed evenly, but not sex, with four women and two men in HUVV, and with five women and two men in HJRJ.

### Ethical approval

The study was approved by the Ethics Committee in Clinical Investigation of North-West Málaga, dated 27 of November 2011, with the Quality Assurance Unit of the Hospital Virgen de la Victoria (Málaga) written authorization for data collection of the patients included in the study, with the previous signing of informed consent. This research was also been approved in Huelva by the Ethics Committee in Investigation of this province in 2015 (PI 008/15).

### Procedure

A member of the research team coordinated each session, supported by an observer. Participants received a process fact sheet in which its different stages were differentiated, from the request for analytical testing until the sample delivery in the laboratory, for information purposes for those participants who were not familiarised with the process. Besides, they received a printed version of the DAFO scheme, where they were asked to give their suggestions and opinions for each item.

Then, comments were shared, keeping the meeting until data saturation was achieved, following the stated scheme according to their own experience on the subject. The opinions could be based on aspects related to their specific competence or those observed in other professional categories involved in the process.

It was not considered mandatory to review all aspects of the SWOT diagram, and interrupting the speech was allowed in the case of questions or clarification on any proposed data.

The session was recorded, and the group coordinator wrote down notes. Both the recording and the notes were transcribed by the observer, focussing on non-verbal communication of the participants. Data saturation was reached after 48 and 46 minutes, respectively.

### Analysis of the information

SWOT analysis is usually considered a final step in strategic analyses. However, in the present study, it was used as a guide for structuring discourse. Qualitative analysis with a phenomenological approach was performed, offering a different approach to the usual one for obtaining participants information. Results were organised by grouping them into the previously defined dimensions of the SWOT structure, which could explain the analysed phenomenon. Thus, each of the stages of this analysis was regarded as a separate dimension.

The number of comments was recorded and posted to establish the importance of speech based on the number of times that the item in question was mentioned. In this regard, the participants' non-verbal language was also taken into account, with notes taken by the observer. The interpretative phenomenological approach was used as a method of discourse analysis.

## Results

The number of comments for each item is described between square brackets. The overview of the session can be seen in Table 1.

**Table 1:** General description of the session.

Issue	Approx. duration
Presentation	3 min
Brief description of SWOT method and handing in of sheet for noting down	5 min
Review of written ideas following the SWOT scheme	15 min
Reading of items by each participant	20 min
Discussion and end of a session	8 min

At the HUVV, the most commented weaknesses were problems when sending carriers through the pneumatic tube [3], together with the difficulties in extending testing in analytical samples already completed or pending [3], and the high number of patients with analytical test requests [2].

Regarding threats, the most mentioned aspect was the possibility of system failure [5], as well as the presence of tubes in poor conditions [2].

The most often described strengths were the digitalisation of the process [2] and the capacity for teamwork [2].

The opportunities identified were the improvement in the management of analytical requests [3] along with the possibility of extending the duration of contracts for temporary staff [2].

At the HJRJ, regarding weaknesses, the most referred comment was problems with the transport of samples by the pneumatic tube [7], together with inadequate conditions for the collection of samples [3] and the number of samples returned by not being well linked [3].

Concerning the threats, the most reviewed aspect was computer problems when there is a failure in Diraya - the hospital network - or the laboratory computer system [7], as well as errors by the excess of analytical requests [4].

The most often described strengths were the digitalising process [2] and the improvement in the delay of the results [2], while detected opportunities were improvement in computer systems [7] along with the ability to improve the transport of samples through the pneumatic tube [7].

There are more reviews (results) than participants, as all of them were invited to freely express their experience, without any limitation in this respect (Table 2 and Table 3).

**Table 2:** University Hospital Virgen de la Victoria participants’ opinions. (LT, laboratory technician; RP, resident physician)

	Professional	Meetings
Weaknesses		
Problems with »target destination« of carriers (fixing origin and destination codes)	Nurse	RP3
Tubes labelling; the present system can result in errors as it requires the previous labelling	Nurse	1
Problems with request scanner	Nurse	1
Possible human errors caused by high workload and stress	Nurse	1
Use of photocopies for request notes	LT	1
Preanalytical errors (wrongly labelled tubes, patient-tube label confusion)	LT	1
Interrupted cold chain	LT	1
New tube labelling system (colour-coded) and size of the barcode	LT	1
Gasometry with air bubbles	Resident Analyst	1
Difficulties in extending testing in analytical samples already completed or pending	Nurse Physician and Resident Analyst	1
Denial of duly justified tests	RP	
High number of requests (greater error probability)	Resident Analyst,	LT
Threats		
System failure	All	5
Presence of tubes in poor conditions	Nurse, LT	2
Higher pressure in response times	LT	1
Strengths		
Teamwork	LT, Resident Analyst	3
Computerised requests	All	4
Pneumatic carriers flow	Nurse, LT	2
Requests extension without any necessary new request	RP	1
Opportunities		
Improving requests management	Nurse, RP	3
Telephone finding of professionals in the case of incidents (improving response times)	Nurse	2
More staff and higher duration of contracts	LT	2
Samples traceability	LT	1

**Table 3:** University Hospital Juan Ramón Jiménez participants’ opinions. (AN, assistant nurse; LT, laboratory technician; CAP, clinical analysis physician; ED, emergency doctor)

		Professional	Dates
*Weaknesses*			
Loss of urine samples, empty tube awaiting sample		Nurse	1
Tests transport through pneumatic tubes, weight, blood cultures		All	7
Network system when the connection fails		Nurse	1
Vacuum failure in coagulation tube		Nurse	1
Sample loss when pneumatic tube failure		AN	1
The arrival of all tubes to the emergency department when there is a failure		AN	1
Samples are returned by not being properly linked (several times)		AN, LT, Physician	3
Errors caused by new staff, staff changes, and high tests requests demand		Physician	2
Difficulties in extending tests requests		Physician	2
Inadequate conditions for sample collection (ammonium, biological fluids, coagulated samples, gasometries with air bubbles)		LT, Physician	3
Illegible requests		LT	1
Sample sending delay		LT, Physician	2
Non-persistent training and information		Physician	1
Outcomes sending delay		Physician	1
* Threats *			
Wrong delivery of samples through a pneumatic tube		Nurse, LT	2
System failures, Software failures with requests linking, wrongly linked or not linked		All	7
Excess of testing requests, errors by the excess of requests		Nurse, Physician, AN	4
Scarce staff resources (ward staff)		Physician	1
Not possible extension of requests due to excess of time since the sample was taken		LT	1
Unjustified samples		Physician	1
*Strengths *			
Ease of sample identification		Nurse	1
Analytical tests linking system		Nurse	1
Quick and safe when the network system is properly working		Nurse, Physician	2
Improvement in outcomes delays		Nurse, Physician	2
Improvement in communication between professionals		LT	1
Better sample traceability when network is properly working		Physician	1
*Opportunities *			
Direct pneumatic tube communication between the emergency department and laboratory		All	3
Resident guidance		Physician	1
Improvement of a communication system (Clinic history software, hospital network)		All	7
Pneumatic tube		All	7

## Discussion

The preanalytical phase is a complex moment within the laboratory process and because of that, it has not been possible to standardise it to date, contrary to what has happened with the analytical phase [8]. This has enabled that, in order to solve the usually high number of errors associated with this phase [9] [10] [11], different approaches have been developed depending on the healthcare scenario in which professionals are involved.

One of the studied environments has been urgent samples and their characteristics. From the now-classic work by Plebani [10], whose data were reviewed by the same researchers ten years later [11], several authors tried to make further progress in the matter and to offer solutions of different types that, somehow, to contribute to control the issue [12] [13] [14].

The existence of different strategies has allowed verifying that it is not a common thing to directly consult with those involved in the process. The validity of this proposal was found in previous experiences in primary care [5] [6]. For this reason, we decided to perform an identical procedure with the professionals involved in the pre-test phase of urgent samples. Although studies have a similar structure, they involved professionals from different areas, so the information obtained was considered as novel and complementary.

Once the focus groups developed, it became clear that availability when judiciously reporting different items was the same in all participants, establishing a flow of information that is considered as of a high utility; identical aspects were identified as relevant by professionals of different categories and centres with, a priori, different points of view.

Aspects related to the weaknesses of the process focused on the problems detected in the transport of samples by the pneumatic tube; in particular, destinations, which proved wrong on many occasions. This problem's resolution is not within direct reach of those involved, unlike the second most mentioned points (difficulties in extending testing in analytical samples already completed or pending [5] in HUVV; sample collection inadequate conditions [3], and the number of samples that are returned for not being well linked [3] in HJRJ), which were addressed by professionals from all areas, starting a brief debate and thus reaching an agreement in principle to solve them.

As for the threats, the system failure was the most stated issue by virtually all the participants. It is without a doubt a logical judgement if we consider the current technological dependence in all health areas, especially in the emergency department [5] [7].

Among the identified strengths, we want to emphasize two of them: first, the sense of teamwork (described both in emergencies and in the laboratory), and secondly, the existence of the computer module of analytical request, which allows streamlining procedures, accelerating results, and facilitating traceability. It is no wonder, therefore, that the possibility of being unable to rely on electronic devices is considered a threat by virtually all professionals.

Finally, participants offered their impressions about opportunities, focusing on three aspects: improving the management of requests and the performance of the pneumatic tube, improving the duration of contracts, especially in the laboratory (very brief in recent months, forcing a repeated training system for new staff), and creating a circuit for professionals localization during the work shift to facilitate potential error solving, as in the case of extending requests, referred above.

On all these points, participants expressed interest in generating groups that interact and allow both implementing proposals and making a follow-up of the same and involving the person responsible for each area.

Although the qualitative approach to corpus analysis has been developed in two hospitals to find similarities between them, and the data obtained finally offered us good information about error sources, the findings cannot be extended to wider populations with the same degree of certainty because the findings of the research are not tested to discover whether they are statistically significant or due to chance. However, this study does allow us to obtain new fields for research and also allow us to explore professionals from a personal perspective. Perhaps the most important aspect of the session, as it happened in other previous studies in the PC area [5] [6] was the general feeling of participants of being heard, and that their suggestions and impressions, if well managed, can help alleviate problems at their work environment, something that is not usually considered by managers. All this is highlighted despite the importance given to active listening provided that, in addition to the scientific aspect, it is necessary to consider the relational aspect and communication skills, especially highly valued in the nursing field [15].

A fundamental purpose of this study is to give a voice to those who are facing health problems and to offer suggestions to managers, even with proposals that facilitate the solution to the problems detected. Their opinions are considered as valuable as those previously given by primary care workers, although they offered a different point of view. This is due to the many differences found when comparing stat labs with routine labs. In addition, the ED staff are also very distinct when compared to primary care staff. The combination of different points of view on similar problems will probably allow us to more easily find solutions. The data obtained have contributed to making visible the risk associated with preanalytical errors, helping to incorporate several corrective measures. Among them, we can find digital test requests, which allow better traceability and decrease the risk of identification errors, as well as informative sessions planning.

## Conflict of interest statement

The authors declare that they have no conflicts of interest in this work.

## List of abbreviations

ED, emergency department; HFMEA, healthcare failure mode and effects analysis; PC, primary care; SWOT, strengths, weaknesses, opportunities, and threats; HUVV, University Hospital Virgen de la Victoria; HJRJ, University Hospital Juan Ramón Jiménez.
